# The diagnostic and prognostic value of real-time ultrasound elastography combined with contrast-enhanced ultrasound parameters for cervical cancer

**DOI:** 10.12669/pjms.41.6.11974

**Published:** 2025-06

**Authors:** Wenfang Cao, Jun Wang, Lina Wang

**Affiliations:** 1 Wenfang Cao, Department of Ultrasound Medicine, Xi’an People’s Hospital, Xi’an Fourth Hospital, Xian, Shaanxi Province 710004, P.R. China; 2 Jun Wang, Department of Ultrasound Medicine, Xi’an People’s Hospital, Xi’an Fourth Hospital, Xian, Shaanxi Province 710004, P.R. China; 3 Lina Wang Department of Ultrasound Medicine, Xi’an People’s Hospital, Xi’an Fourth Hospital, Xian, Shaanxi Province 710004, P.R. China

**Keywords:** Real-time tissue elastography, Contrast-enhanced ultrasound, Parameters, Cervical cancer, Diagnostic, Prognostic

## Abstract

**Objective:**

This study aimed to explore the diagnostic and prognostic value of real-time tissue elastography (RTE) and contrast-enhanced ultrasound (CEUS) parameters in cervical cancer (CC).

**Methods:**

This retrospective study included RTE and CEUS records of 144 patients who underwent cervical ultrasound in Xi’an People’s Hospital from January 2022 to August 2023. Patients were grouped into the malignant group and the benign group based on the characteristics of the cervical lesions. The RTE and CEUS parameters were compared between the groups and between patients with different pathological features and prognoses within the malignant group. The Receiver Operating Characteristic (ROC) curve was used to analyze the predictive value of RTE and CEUS parameters for the prognosis of patients with malignant CC.

**Results:**

The elasticity score, strain ratio (SR), time to peak (TTP), peak intensity (PI), and area under the curve (AUC) of the malignant group were higher than those of the benign group (P<0.05). There were significant differences in the levels of RTE and CEUS parameters among patients with different International Federation of Gynecology and Obstetrics (FIGO) stages and pathological grades (P<0.05). The RTE and CEUS parameter levels positively correlated with the FIGO staging and pathological grading of cervical cancer (P<0.05). The elasticity score, TTP, PI, SR, and AUC of patients with poor prognosis were higher than those with good prognosis (P<0.05). The combined predictive efficacy of RTE and CEUS parameters for the prognosis of CC was superior to their individual predictions.

**Conclusions:**

The combination of RTE and CEUS parameters of CC patients are closely related to the pathological characteristics and prognosis of the disease.

## INTRODUCTION

Cervical cancer (CC) is the fourth most common cause of cancer death in women of all ages after breast, lung, and colorectal cancer.^1^–^3^ Early-stage CC lacks specific clinical manifestations, and abnormal vaginal secretions, bleeding, and other symptoms are easily confused with other gynecological diseases, making the diagnosis difficult.^2^,^3^ The current treatment of CC advocates personalized treatment, and a comprehensive evaluation of the patient is required before determining the treatment plan.^4^,^5^ Moreover, evaluating the efficacy of cervical cancer treatment can have significant implications for developing clinical follow-up plans.^5^

Ultrasonic elastography is an important imaging diagnostic technique in clinical practice.^6^ It is based on examining tissue hardness to distinguish the benign and malignant nature of diseases.^6^,^7^ Real-time ultrasound elastography (RTE) uses a focused ultrasound beam to create shear waves in viscoelastic tissue and aims to quantitatively image the Young’s E modulus, providing an objective reference for disease diagnosis and evaluation.^6^–^8^ Contrast-enhanced ultrasound (CEUS), another important diagnostic measure for CC,^9^ mainly involves injecting microbubble contrast agents into target tissues or organs to increase blood flow and peripheral tissue echoes by enhancing backscatter, altering acoustic attenuation, etc. CEUS, therefore, can be used to distinguish between lesions and surrounding normal tissues.^9^,^10^

Currently, there is limited evidence of the clinical value of RTE combined with CEUS in evaluating CC. This study retrospectively analyzed the imaging data of cervical cancer patients who underwent RTE and CEUS to explore the relationship between RTE and CEUS parameters and the diagnosis and prognosis of cervical cancer patients.

## METHODS

This retrospective study included RTE and CEUS imaging records of patients who underwent cervical ultrasonography in Xi’an People’s Hospital from January 2022 to August 2023. Patients were grouped into the malignant group and the benign group based on the characteristics of the cervical lesions.

### Ethical approval:

Patient informed consent was waived, and the study was approved by the institution’s ethics committee (Approval number: KJLL-Z-K-2024072, Date: December 6, 2024).

### Inclusion criteria:

- Patients met the diagnostic criteria for cervical cancer or cervical intraepithelial neoplasia.^11^
- Both benign and malignant lesions were diagnosed through biopsy or surgical pathology.
- Complete preoperative imaging and clinical data.
- Cervical cancer patients have complete follow-up imaging data at six months after surgery and have been followed up for more than one year.

### Exclusion criteria:

- Presence of other malignant tumors.
- Patients with infectious diseases and endocrine system diseases.
- Breastfeeding and pregnant women.

### RTE:

The imaging was performed using Philips EPIQ Elite W-type color Doppler ultrasound diagnostic instrument and C10-3v intracavity probe. The probe frequency was set to 3-10 MHz. The patient was instructed to empty the bladder and lay in the bladder lithotomy position. A probe was inserted into the vagina for routine ultrasound examination, selecting the area with the most explicit lesion presentation effect. The operator then switched to elastic imaging inspection, making two real-time dynamic images with two-dimensional images as reference and observing the corresponding elastic images while ensuring the selection range of lesion images as much as possible and ensuring the optimal sampling position and image clarity for ultrasound elasticity presentation.

The dynamic images were saved and observed, using the lesion and surrounding tissue (5- mm) as the region of interest (ROI), and the elasticity score was recorded. Elastic scoring criteria: One score: if most lesions are green, score one point; Two scores: There is a small amount of blue and green area in the middle of the lesion, which is greater than 90%; Three scores: mixed blue-green color within the lesion, with similar proportions; Four scores: Most lesions are blue with a small amount of green around them; Five scores: the lesion is mostly blue. The ratio of elastic strain rate (SR) recording: When the volume and shape of the lesion were clear, the sampling box was expanded to ensure that it can completely cover the lesion area; A frame was drawn around the lesion and surrounding normal cervical tissue at the same level (within a 5mm range), and the SR value was automatically obtained.

### CEUS:

The operator switched to contrast mode after elastography examination. The ultrasound contrast agent Sonovir (Bracco company, Italy) was dissolved in 5 ml of physiological saline to prepare a suspension and 2.4 ml of contrast agent suspension was quickly injected through the elbow vein. The optimal section for blood flow perfusion was selected. The operator switched to low mechanical index CEUS mode, started synchronous timing, and obtained real-time 2D and CEUS images. The advance and retreat of the contrast agent in the lesion were dynamically monitored, and the imaging video was saved after 180 seconds. A frame was drawn around the lesion and normal tissue at the same level based on the perfusion characteristics of the contrast agent in the lesion area and the way the contrast agent enters and exits the lesion. One-click automatic acquisition of the time-signal intensity (TIC) curve, recording contrast parameters including time to peak (TTP), peak intensity (PI), and area under the curve (AUC) were done.

### Observation indicators:

- Baseline data, including age, body mass index (BMI), marital status, and education level.
- Preoperative RTE and CEUS parameters, including elasticity score, SR, TTP, PI, and AUC.
- Different pathological features, including FIGO staging and pathological grading.
- The elasticity score, SR, TTP, PI, and AUC levels of the malignant group were rechecked at six months after surgery.
- The prognosis of patients in the malignant group one year after surgery is divided into good prognosis for survivors and poor prognosis for those who die or have disease recurrence.

### Statistical analysis:

The data were input into Microsoft Excel and analyzed using SPSS version 26.0 (IBM Corp, Armonk, NY, USA). According to the Shapiro-Wilk test, the distribution normality was evaluated. Normal distribution data were represented by mean ± standard deviation (SD), and an independent sample t-test was used to compare the two groups.

The comparison between different FIGO stages and pathological grades was conducted using a one-way analysis of variance (ANOVA). Non-normally distributed data were represented by median and interquartile range, and the Whitney U test was used for comparison between the two groups. Kruskal Wallis H test was used to compare different FIGO stages and pathological grades. Spearman was used to analyze the correlation between pathological features (FIGO staging, pathological grading) of cervical cancer and RTE and CEUS parameters. The ability of RTE and CEUS parameters to predict the prognosis of cervical cancer was assessed using receiver operating characteristic (ROC) curves. A p-value less than 0.05 was considered statistically significant. All reported p-values were two-sided.

## RESULTS

This study included and retrospectively analyzed records of 144 patients. Of them, 89 were diagnosed with the malignant lesion (the malignant group), and 55 patients with benign lesions comprised the benign group. There was no significant difference in the clinical characteristics such as age, BMI, marital status, and education level between the two groups of patients (P>0.05) (Table-I). The elasticity score, SR, TTP, PI, and AUC levels in the malignant group were significantly higher than those in the benign group (P<0.05) (Table-II).

**Table-I:** Comparison of clinical characteristics between two groups.

Characteristics	Malignant group (n=89)	Benign group (n=55)	t/χ^2^	P
Age (years), Mean±SD	53.84±7.51	52.31±8.43	1.135	0.258
BMI (kg/m^2^), Mean±SD	23.68±3.28	24.24±3.16	-1.024	0.308
*Marital status, n (%)*			0.168	0.682
Married	69 (77.5)	41 (74.5)		
Unmarried/divorced	20 (22.5)	14 (25.5)		
*Educational level*			1.593	0.207
Junior high school and below	64 (71.9)	34 (61.8)		
High school and above	25 (28.1)	21 (38.2)		

**Table-II:** Comparison of Relevant Parameters between Malignant and Benign Groups, M (P25/P75).

Parameters	Malignant group (n=89)	Benign group (n=55)	Z	P
Elasticity score	3 (2-4)	2 (1-2)	-6.828	<0.001
SR	3 (2-4)	2 (2-3)	-5.483	<0.001
TTP (second)	36 (35-42)	28 (25-32)	-7.65	<0.001
PI (dB)	27 (25-30)	23 (20-25)	-6.907	<0.001
AUC	2545 (2325-2654)	2154 (1987-2401)	-5.433	<0.001

Among 89 patients with malignant tumors, 16 had FIGO stage II, 57 were stage III, and 16 were stage IV. Regarding the different pathological grades, 19 patients were classified as G1, 57 as G2, and 13 as G3. There were significant differences (P<0.05) in the levels of elasticity score, SR, TTP, PI, and AUC among patients with different FIGO stages and pathological grades (Table-III). The Spearman analysis results showed that elasticity score, SR, TTP, PI, and AUC significantly positively correlated with FIGO staging and pathological grading of CC (P<0.05) (Table-IV).

**Table-III:** Comparison of relevant parameters among patients with different pathological features.

Pathological features	Classification	n	Elasticity score, M(P25/P75)	SR, M(P25/P75)	TTP (s), Mean±SD	PI (dB), Mean±SD	AUC, M(P25/P75)
FIGO	Ⅱ	16	2 (2-3)	2 (2-3)	33.00±4.20	24.31±2.18	2323 (2050-2483)
	Ⅲ	57	3 (2-4)	3 (2-4)	38.04±5.43	27.51±3.24	2546 (2325-2658)
	Ⅳ	16	4 (4-5)	4 (4-5)	41.56±5.77	31.31±3.79	2658 (2591-2770)
	*F/H*		30.704	25.897	10.647	19.399	22.130
	*P*		<0.001	<0.001	<0.001	<0.001	<0.001
Pathological grading	G1	19	2 (2-3)	2 (2-3)	33.68±4.22	24.47±2.52	2321 (2054-2453)
	G2	57	3 (3-4)	3 (2-4)	37.88±5.49	27.65±3.09	2548 (2351-2654)
	G3	13	4 (4-5)	4 (4-5)	43.23±5.02	32.08±3.80	2685 (2653-2841)
	*F/H*		30.102	25.064	13.141	23.389	28.491
	*P*		<0.001	<0.001	<0.001	<0.001	<0.001

**Table-IV:** Spearman analysis.

Item		Elasticity score	SR	TTP	PI	AUC
FIGO	*r*	0.520	0.508	0.447	0.547	0.501
	*P*	<0.001	<0.001	<0.001	<0.001	<0.001
Pathological grading	*r*	0.471	0.502	0.486	0.563	0.568
	*P*	<0.001	<0.001	<0.001	<0.001	<0.001

**Fig.1:**
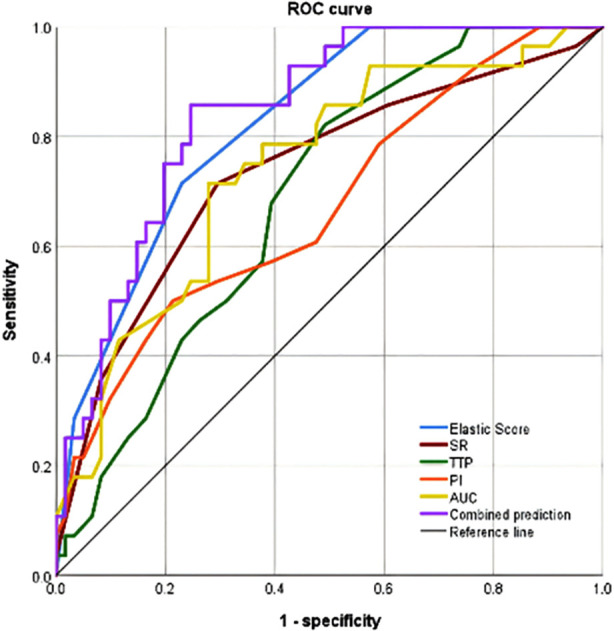
ROC curve.

Among 89 patients with malignant tumors, 61 had a good prognosis, and 28 had a poor prognosis. The elasticity score, SR, TTP, PI, and AUC levels of patients with poor prognosis were significantly higher than those with good prognosis (P<0.05) (Table-V). The ROC curve analysis showed that the combination of elasticity score, SR, TTP, PI, and AUC had significantly better predictive efficacy for the prognosis of cervical cancer than each indicator alone (Table-VI) (Fig.1).

**Table-V:** Comparison of relevant parameters among patients with different prognostic conditions.

Prognosis	n	Elasticity score, M(P25/P75)	SR, M(P25/P75)	TTP (s), Mean±SD	PI (dB), Mean±SD	AUC, M(P25/P75)
Poor	28	4 (3-5)	4 (3-5)	40.25±5.25	29.39±4.13	2653 (2546-2693)
Good	61	3 (2-3)	3 (2-4)	36.62±5.80	26.80±3.36	2453 (2315-2652)
*t*		9.367	6.582	2.820	3.136	13.734
*P*		<0.001	<0.001	0.005	<0.001	<0.001

**Table-VI:** The predictive value of RTE and CEUS parameters for patient prognosis.

Indexes	AUC	Sensitivity (%)	Specificity (%)	95%CI
Elasticity score	0.824	71.4	77.0	0.739~0.910
SR	0.737	71.4	70.5	0.620~0.854
TTP	0.689	82.1	50.8	0.579~0.799
PI	0.673	50.0	78.7	0.552~0.795
AUC	0.743	71.4	72.1	0.633~0.853
Combined predictive	0.843	85.7	75.4	0.762~0.924

## DISCUSSION

This study demonstrated that the combination of RTE and CEUS parameters is highly effective in distinguishing cervical cancer from cervical intraepithelial neoplasia. The elasticity score, SR, TTP, PI, and AUC of patients with malignant cervical lesions were significantly higher than those with benign lesions. These results are consistent with the previous observations. Guo Haixia et al.^12^ found that combining CEUS technology with conventional ultrasound diagnosis can improve the accuracy of lesion qualitative judgment. Sui et al.^13^ also found that combining CEUS and RTE significantly improved the diagnostic ability to distinguish malignant and benign thyroid nodules compared to using CEUS or RTE alone. It is plausible that the improved diagnostic performance of the combined method may be due to the ability of RTE to reflect the hardness of tissues.

Statistical studies such as the study by Dudea Simon M et al.^14^ have confirmed that the hardness of cervical cancer tissue in elastography is significantly higher than that of benign lesions, mainly due to changes in tissue structure caused by the infiltration and proliferation of cancer cells. Therefore, the increase in elasticity score and SR can be an important feature of CC.^12^,^13^,^15^ At the same time, the increase in the CEUS parameters TTP, PI, and AUC reflects the blood supply characteristics of CC tissue, the abundant blood supply of which may be closely related to tumor growth and progression.^13^,^16^ Ignat RM et al.^17^ analyzed the application value of CEUS in cervical cancer, demonstrated differences in blood flow perfusion and other characteristics between patients with benign and malignant cervical lesions, and suggested that cervical cancer patients have abundant neovascularization and a large number of arteriovenous fistulas, with fast blood flow velocity. This observation is consistent with the viewpoint of this study. This study revealed significant differences in elasticity score, SR, TTP, PI, and AUC among cervical cancer patients with different FIGO stages and pathological grades. As FIGO staging and pathological grading increased, elasticity score, SR, TTP, PI, and AUC also increased accordingly, consistent with the pathological and physiological characteristics of CC.^2^,^3^ Studies have demonstrated that cancer cells continue to proliferate and invade during the progression of tumors, leading to increased tissue hardness and richer blood supply.^18,19^ Spearman correlation analysis confirmed that elasticity score, SR, TTP, PI, and AUC positively correlated with FIGO staging and pathological grading. This observation further emphasizes the importance of the above parameters in assessing the severity of cervical cancer.

Imaging, such as ultrasonography and magnetic resonance imaging (MRI), is crucial for evaluating the efficacy of CC treatment. MRI has a high soft tissue resolution and multi-parameter imaging capabilities that can evaluate the residual CC tissue after the treatment.^20^ However, MRI has some disadvantages due to the relatively high price, long examination time, and susceptibility to metal artifacts.^21^ In recent years, the application of ultrasound for non-invasive monitoring of tumor hyperthermia has gradually become a clinical hotspot.^22^ The results of this study showed that cervical cancer patients with poor prognosis one year after surgery had higher levels of elasticity score, SR, TTP, PI, and AUC at the 6-month follow-up after the surgery compared to patients with good prognosis. This result is consistent with previous research findings^23,24^ and suggests that RTE and CEUS parameters not only reflect the current status of cervical cancer but also have prognostic value. This study conducted ROC curve analysis, showing that the joint prediction value of RTE and CEUS indicators was more effective than that of individual predictions.

Similarly to these observations, Fang Wentao et al.^23^ showed that ultrasound elastography and CEUS could be used for the pre-diagnosis and postoperative efficacy evaluation of breast nodule microwave ablation. The quantitative parameter values significantly correlated with the ablation effect and could serve as auxiliary reference indicators for ablation efficacy, postoperative recurrence, and residual lesion detection. The observed efficiency of the combined RSE/CEUS imaging may be explained by the fact that RTE estimates the corresponding conditions within the tissue and evaluates changes in the internal structure of the tissue or lesion by obtaining differences in mechanical properties, such as elastic modulus.^14^,^16^ Meanwhile, CEUS uses hemodynamic changes as a criterion for distinguishing between benign and malignant lesions and can clearly display the distribution of lesions and their surrounding blood vessels by increasing blood flow Doppler signals through contrast agents. It is not affected by blood flow direction, velocity, or vessel diameter.^17,24^

So far, the clinical utility of the combination of RTE and CEUS for CC have not been carefully accessed. To fill this gap of knowledge, we conducted this retrospective study and found that comprehensive consideration of multiple ultrasound parameters can more comprehensively and accurately evaluate the prognosis of patients, providing stronger support for clinical treatment decisions.

### Limitations

This is a retrospective study with a relatively small sample size, which may impact the generalizability of the results. Secondly, due to factors such as lesion volume and blood vessels, the combination of RTE and CEUS examination may still result in misdiagnosis. Thirdly, the results of RTE and CEUS examinations are influenced by various factors, such as the operator’s proficiency and surrounding intestinal distension, which can affect the application of ultrasound examination technology in cervical diseases. Finally, a comprehensive judgment based on the patient’s clinical manifestations, other imaging examinations, and pathological results is needed in real-life clinical practice.

## CONCLUSION

The combination of RTE and CEUS parameters have an important application value in the diagnosis, disease assessment, and prognosis prediction of CC, providing new ideas and methods for the clinical management of cervical cancer. Further studies are needed to improve and optimize the application of this combined imaging method.

### Authors’ contributions:

**WC:** Literature search, study design and manuscript writing, revision and validation. **JW and LW:** Data collection, data analysis and interpretation. Critical Review. All authors have read, approved the final manuscript and are responsible for the integrity of the study.
